# Thermicity of the Decomposition of Oxygen Functional Groups on Cellulose-Derived Chars

**DOI:** 10.1021/acsomega.2c07429

**Published:** 2022-12-15

**Authors:** Christin Pflieger, Till Eckhard, Gunnar Schmitz, Vanessa Angenent, Maximilian Göckeler, Osvalda Senneca, Rochus Schmid, Francesca Cerciello, Martin Muhler

**Affiliations:** † Laboratory of Industrial Chemistry, Ruhr University Bochum, 44801Bochum, Germany; ‡ Computational Materials Chemistry Group, Ruhr University Bochum, 44801Bochum, Germany; § Istituto di Scienze e Tecnologie per l’Energia e la Mobilità Sostenibili, Consiglio Nazionale delle Ricerche, 80125Napoli, Italy

## Abstract

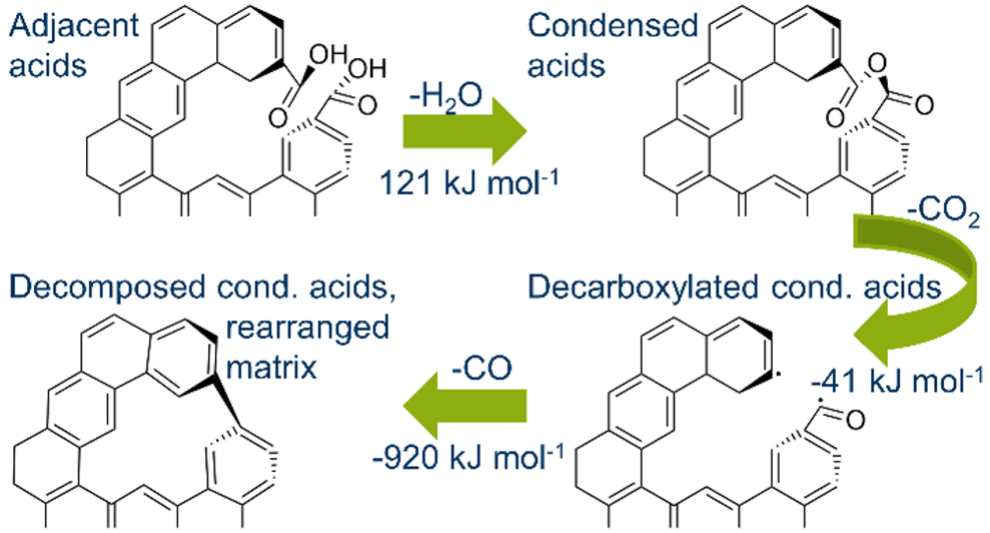

The evolution of oxygen functional groups (OFGs) and the associated thermic effects upon heat treatment up to 800 °C were investigated experimentally as well as by theoretical calculations. A synthetic carbon with a carbonaceous structure close to that of natural chars, yet mineral-free, was derived from cellulose and oxidized by HNO_3_ vapor at different temperatures and for varied durations in order to generate char samples with different concentrations and distributions of OFGs. The functionalized samples were subjected to calorimetric temperature-programmed desorption measurements in correlation with an extensive effluent gas analysis, thereby focusing on the specific heat effects of individual OFG evolution. Interpretation of the experimental results was aided by density functional theory (DFT) calculations which allowed one to infer the thermal stability of different OFGs and the reaction energy associated with their evolution upon heating. Results showed that, with increasing temperature, H_2_O was released due to the loss of physisorbed water, the decomposition of clusters bound to carboxylic acids, and condensation reactions. The associated heat uptake amounted to about 100 kJ mol^–1^. Contrarily, the release of CO_2_, attributed to the decomposition of condensed acids, carboxylic acids, anhydrides, and lactones, resulted in a heat release of about 40 kJ mol^–1^. The most strongly pronounced thermic effects were detected for the release of CO, comprising highly exothermic effects due to the decomposition of condensed acids and carbonyls/quinones as well as endothermic effects attributed to anhydrides and phenols/ethers. Notably, anhydrides can be formed during the oxidative treatment as well as during heating by condensation of adjacent carboxylic acids. In the latter case, the two-step decomposition is overall highly exothermic, indicating the associated occurrence of pronounced carbon matrix rearrangements. DFT investigations suggest that these rearrangements not only affect the immediate OFG proximity but also involve several carbon sheets.

## 1 Introduction

Solid carbons, especially those derived from biomass, are gaining increasing interest in chemical and materials engineering for energy-related and environmental applications including the development of sorbents, catalysts, and materials for electronics as well as utilization as CO_2_-neutral substitutes of coal in processes of combustion, oxycombustion, and gasification. Important features of these materials are porosity, thermal and electric conductivity, and combustion and gasification reactivity. The nature and thermal stability of the oxygen functional groups (OFGs) which form when a carbon surface gets in contact with an oxidizing agent^1,2^ have also very important effects on the surface properties and are critical for the potential utilization of the biochars in the different mentioned applications:^3,4^

- OFGs contribute to the wetting and adsorption behavior, introducing hydrophilic sites on the intrinsically hydrophobic carbon surface.^5,6^
- Using carbon materials as catalyst support, OFGs provide anchoring sites for metals and ultimately affect their dispersion, morphology, and sintering propensity.^7,8^
- OFGs act as active sites in carbon-catalyzed reactions like wet air oxidation^9^ and nitrobenzene reduction^10^ as well as enhance the capacitance of electrochemical materials.^11^
- The formation of OFGs through chemisorption of molecular oxygen and successive evolution until the release of CO_*x*_ are at the basis of combustion and gasification kinetics.^12−14^

The OFGs on the surface of carbon materials depend on the carbon matrix they are formed on^15,16^ and have been the object of several research papers over the last decades. Titration methods^11,17,18^ have been used to characterize OFGs. Notably this technique may underestimate the total amount of OFGs as only acidic and basic groups are accounted for, whereas the neutral ether and carbonyl groups cannot be detected.^19^ More recently, indeed, X-ray photoelectron spectroscopy (XPS) and temperature-programmed desorption (TPD) have been used to investigate OFGs present on carbon materials.^20,21^ Recently, some experimental works have tried to combine the analysis of the carbon surface by XPS with thermoanalytical techniques (TPD and differential scanning calorimetry (DSC)) in order to investigate the thermochemistry of the main steps of the carbon–oxygen reaction^22−25^ in combustion processes. Levi et al.^25^ and Cerciello et al.^24^ within the theoretical frame of the three-step semilumped kinetic model of coal combustion proposed by Hurt and Niksa^26,27^ suggested that the overall thermicity of a carbon–oxygen reaction results from the sum of exothermic (chemisorption and surface oxide rearrangement) and endothermic (CO_*x*_ abstraction) steps.^25^ Accordingly, while the overall combustion heat would be always exothermic, the heat registered in a TPD step could be either endo- or exothermic^22^ depending on the operating temperature, as it results from the balance of the exothermic stabilization of metastable oxides and the endothermic desorption of CO_*x*_.

As noted, the above works referred to lumped reaction steps, developed in the context of coal combustion science, and were not able to resolve the heat effects originating from individual OFGs due to the high heating rates applied^22,25^ or because these were hidden by the overall oxidation reaction.^23,25^ Furthermore, these studies using natural chars may be strongly affected by contained minerals which are known to exhibit pronounced catalytic effects on carbon surface reactions.^23,28^

Ab initio methods provided by computational chemistry can also be very useful to investigate the thermicity of generation, transformation, and decomposition of OFGs. Computational chemistry has been successfully applied to investigate the stability of OFGs residing on relatively simple carbon materials, such as graphenes,^29−32^ and demonstrated that, subsequent to their formation, OFGs undergo surface reactions, interact, and evolve into different OFGs. Application of computational chemistry to complex carbonaceous structures such as that of biomass chars appears much more complicated, even though the pioneering work by Mathews et al.,^33−35^ who applied computational chemistry to describe the complex carbon structure of coal, paved the way toward further development of computational models of natural carbons.

In the present work, in order to separate the effects of mineral matter while keeping the complexity of the carbon structure typical of natural chars, a model synthetic carbon was purposely produced from cellulose according to a previously developed two-step procedure, comprising hydrothermal carbonization followed by pyrolysis.^36^ As shown in previous work, this model synthetic carbon material is characterized by a complex structural order comparable to that of a natural coal char, while being inherently mineral-free. Low heating rate calorimetric TPD measurements up to 800 °C coupled with gas analysis allowed one to obtain quantitative insights into the decomposition processes for individual OFGs without any catalytic influences and with increased resolution of thermic effects. The experimental work has been coupled with DFT calculations using the Turbomole program package^37^ version 7.5,^38^ employing the ridft module in order to assist in the interpretation of experimental results. This novel approach provides thermochemical parameters for OFG evolution of mineral-free carbons with complex structures, useful for validation and development of models. This work represents a step forward toward understanding and modeling the oxidative and thermal processes of natural carbon materials characterized by both complex three-dimensional structure and the presence of catalytically active mineral phases.

## 2 Experimental Section

### 2.1 Materials: Production and Functionalization of Synthetic Carbon

A mineral-free synthetic carbon was produced from microcrystalline cellulose (125 to 200 μm, VIVAPUR by JRS Pharma) through a hydrothermal carbonization step, followed by a pyrolysis step. The combination of the two techniques has the advantage of optimizing both the yield of solid product, which would have been very scarce for direct cellulose pyrolysis, and its structural order, which would have been hardly developed in the case of HTC alone.

For the hydrothermal carbonization, a polytetrafluoroethylene inliner of a 500 mL stainless steel autoclave was loaded with a suspension of 60 g of microcrystalline cellulose and 300 mL of HPLC-grade H_2_O. Heating the sealed autoclave to 200 °C for 24 h resulted in an autogenous pressure of about 1.55 MPa due to the temperature increase. Afterward, the solid residue was filtered, washed with HPLC-grade H_2_O until a neutral pH value was reached, and then dried overnight at 105 °C resulting in a synthetic hydrochar. Low heating rate pyrolysis was performed subsequently in several batches by loading each time about 1 g of the hydrochar in a quartz boat placed inside a horizontal tube furnace. Heating in 70 mL min^–1^ flowing N_2_ (99.999% purity) to 800 °C with 5 °C min^–1^ and holding this temperature for 120 min resulted in the completely devolatilized synthetic char, labeled “MH800”.

MH800 was further oxidized by HNO_3_ vapor treatments. This treatment was chosen since it combines the advantages of wet and dry processing. For comparison, also an oxidation treatment in air at 200–450 °C has been carried out, as reported in the Supporting Information (Section S1), and results confirmed that oxidation by HNO_3_ is more efficient than oxidation by molecular oxygen, enabling a high degree of functionalization while concurrently minimizing carbon mass loss.^39^

The procedure for oxidation by HNO_3_ vapor was the following: 1 g of MH800 was placed into a flow-through quartz crucible inside a vertical quartz tube and heated to temperatures ranging from 125 to 200 °C. Below the crucible, a round-bottom flask containing HNO_3_ (≥65%) was heated to 125 °C in an oil bath, thereby inducing boiling of HNO_3_. The resulting vapor passed through the char material before it was condensed by a H_2_O-cooled condenser. Liquid HNO_3_ dropped back directly into the round-bottom flask without any contact to the char. The duration of the vapor phase treatment was varied in the range of 0.5 to 24 h. The char samples oxidized by HNO_3_ vapor were labeled as “O-MH800-*t*_ox_-*T*_ox_” with *t*_ox_ as the duration of the oxidation treatment. For example, MH800 treated for 0.5 h at 125 °C in HNO_3_ vapor was denoted as “O-MH800-0.5-125”.

### 2.2 Characterization

Compositional analysis of the char samples was performed using a CHNS elemental analyzer (vario MICRO cube by Elementar Analysensysteme). As the reagent cellulose is composed of CHO and only N could have been implemented as an additional elemental component during pyrolysis in N_2_ and functionalization by HNO_3_ vapor, the samples were inherently free of S and minerals, enabling the calculation of the O weight fraction *w* as a difference:
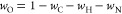
1Samples were further analyzed by XPS measurements of the C 1s and the O 1s regions as described in the Supporting Information (Section S2.2).

### 2.3 TPD-EGA and TPD-Calorimetric Measurements

TPD measurements were performed with an extensive evolved gas analysis (EGA) quantifying H_2_O, CO_2_, and CO in a flow setup while heating the sample with 5 K min^–1^ up to 800 °C to resolve different OFG contributions. Analogously, calorimetric TPD measurements were performed in a Tian-Calvet type calorimeter implemented in a flow setup. The detailed setup specifications and reaction conditions are given in the Supporting Information (Sections S2.3–S2.5) along with the detailed description of the analysis of determined effluent gas and heat flow curves by deconvolution into Gaussian contributions based on previous literature.^40^ The assignment of EGA peaks to different OFGs is discussed in Section 3.2. The parameters obtained from this deconvolution were then used to deconvolute the heat flow curves of the calorimetric TPD experiments, in which the contributions of different OFGs were not individually resolved.

## 3 Results and Discussion

### 3.1 Characterization of the Differently Functionalized Chars

The elemental compositions of the investigated chars are summarized in Table 1.

**Table 1:** Elemental Compositions (*w*, wt %) of the Char Samples (Sulfur- and Mineral-Free; N May Result from Pyrolysis in N_2_ or Functionalization by HNO_3_ Vapor)

Sample	*w*_C_	*w*_H_	*w*_N_	*w*_O_^a^
MH800	95.5	0.8	0.0	3.7
O-MH800-0.5-125	82.9	1.8	0.5	14.8
O-MH800-1-125	79.5	1.6	0.8	18.0
O-MH800-1-200	72.1	1.5	1.0	25.4
O-MH800-6-200	55.7	1.5	1.5	41.3
O-MH800-6-150	61.2	1.8	1.0	36.1
O-MH800-6-125	63.7	1.9	0.9	33.6
O-MH800-12-125	62.6	2.0	1.0	34.4
O-MH800-18-125	53.5	1.8	1.1	43.6
O-MH800-24-125	54.5	2.0	0.0	43.5

a Calculated as difference (eq 1).

Starting from MH800 with a high carbon content of about 96% and only 4% oxygen, both oxidation with molecular oxygen and the HNO_3_ vapor treatments up to 1 h led to a maximum of 25% oxygen content. Contrarily, the extended (≥6 h) HNO_3_ vapor treatments led to a strongly increased oxygen content of up to 44%. Such high percentages of oxygen were desired in the current experimental work in order to better investigate the evolution of OFGs, even though they are not typical of realistic combustion conditions.

**Figure 1:**
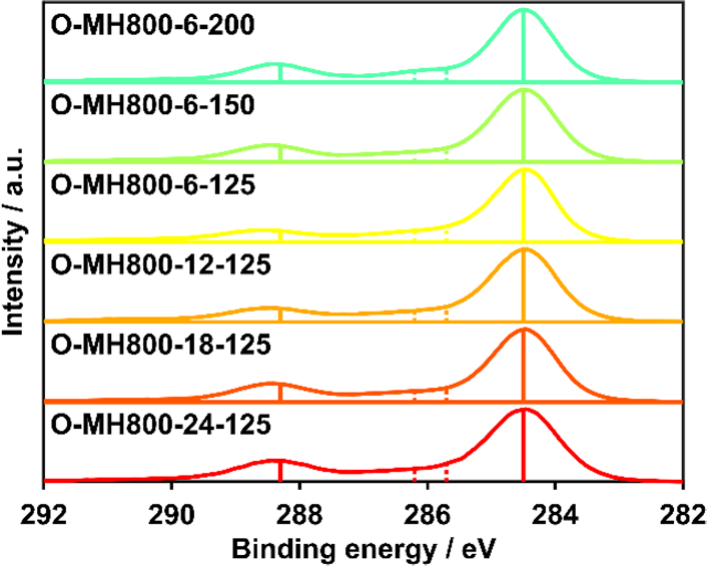
Normalized XPS spectra (C 1s region) of the six most intensely functionalized samples, with solid vertical lines indicating visible peaks and the dashed lines indicating further expected peak positions.

XPS measurements for samples with different degrees of functionalization are shown in Figure 1.

In the C 1s spectra, there are two clearly visible peaks around 284.5 and 288.3 eV. These peaks can be assigned to graphitic carbon and carboxylic functional groups based on first-principles calculations.^41^ Notably the intensity of the 284.5 eV peak and its relative constancies for differently oxidized samples confirms the attainment of a good degree of structural order compatible with that of turbostratic carbons and the negligible effect of the oxidative treatment on the carbonaceous structure. At the same time the presence of the 288.3 eV peak recalls the features of biochars and traces back to cellulose used as material precursor. The O 1s spectra are not reported since the resolution of the XPS instrument used for current analysis turned out to be too low to appreciate all of the relevant peaks, which were otherwise detected in previous work using synchroton radiations.^41^

### 3.2 Quantification of OFGs by TPD-EGA Measurements

**Figure 2:**
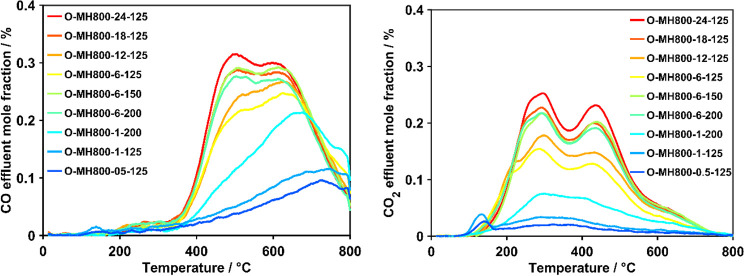
Comparison of evolving CO_*x*_ (left: CO, right: CO_2_) for the differently functionalized samples.

Remarkable differences were observed in the shapes and intensities of the CO_*x*_ EGA curves of char samples with different oxygen contents (Figure 2).

**Figure 3:**
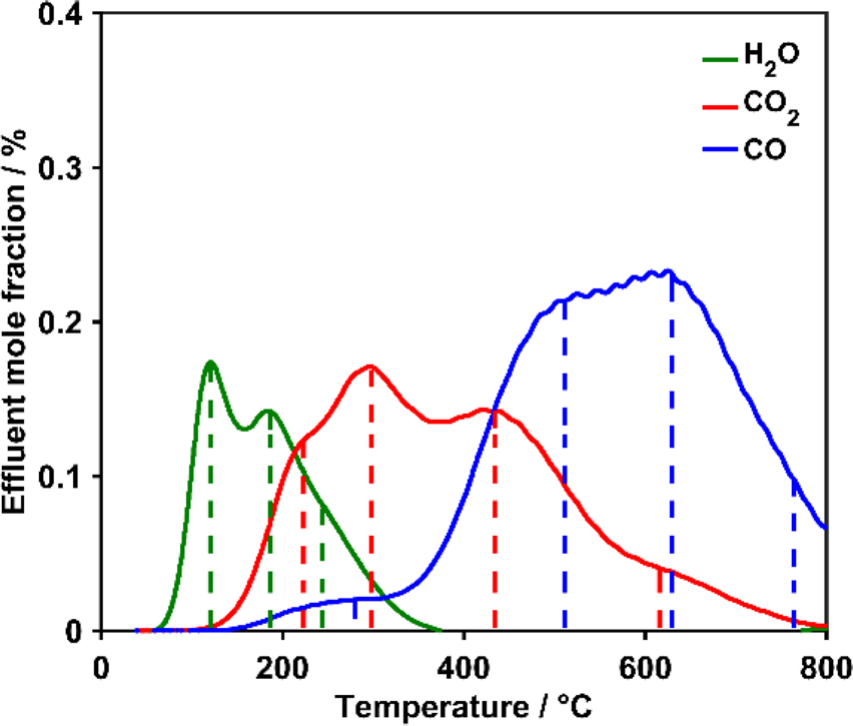
TPD-EGA curves of O-MH800-12-125 with marked local maxima and shoulders.

For the less functionalized samples with a content below 30%, the resolution of CO_*x*_ EGA and heat flow curves (Figure S3.1) was low and did not allow a reliable deconvolution. Therefore, extensive effluent gas quantification and the subsequent deconvolution procedure were applied to the six best resolved curves, which were the ones derived from the samples extensively functionalized for at least 6 h using HNO_3_ vapor. The number of distinguishable contributing peaks to the H_2_O, CO_2_, and CO curves was estimated to amount to three in the case of H_2_O and four in the case of CO_*x*_ (Figure 3).

A uniform TPD-EGA deconvolution based on preset parameters was impaired by the individual temperature ranges of OFG decomposition, depending on sample properties as well as TPD conditions.^5,21^ Nevertheless, there is an agreement on general stability trends for the CO_*x*_ release upon OFG decomposition.^11,42,43^ Carboxylic acids releasing CO_2_ upon decomposition are reported to be the least stable groups, followed by anhydrides releasing both CO_2_ and CO. Lactones are the most stable CO_2_ releasing groups, whereas CO release can be attributed to two additional contributions. Due to their similar properties with regard to temperature stability, decomposition product, and type of carbon–oxygen bond, a differentiation of phenols and ethers as well as of carbonyls and quinones as origin for these two high-temperature CO peaks is not possible.^15^ The assignment listed so far accounts for three contributing peaks in each CO and CO_2_ curve. However, many researchers observed additional low-temperature evolution of CO and CO_2_.^20,44^ The combined CO_*x*_ evolution can be explained by the condensation of adjacent carboxyl groups upon heating, forming additional anhydride-type OFGs, the so-called condensed acids.^3,5^ Condensed acids are assumed to differ from anhydrides formed by the prior functionalization treatment, therefore being less thermally stable and decomposing at lower temperatures than the previously listed OFGs.^16^ Other condensation reactions like the generation of lactones from phenols and carboxylic acids or of ethers from adjacent phenols may be possible in addition.^20,42^ For H_2_O, physisorbed molecules^21,45^ as well as molecules forming clusters bound by hydrogen bridges to carboxylic acid groups^17,46^ are assumed to be present on carbon materials. Further, H_2_O release at higher temperatures occurring in the same range as the two-step decomposition of condensed acids was reported as characteristic for H_2_O released from condensation reactions.^15,16^ According to the general TPD-EGA curve assignment, dehydration results in condensed acids that are destabilized and directly release CO_2_, whereas CO desorbs subsequently, which is typical for anhydrides.^15,18^ Based on these reported trends on relative thermal stability, the EGA curves obtained in this work were interpreted. The assignment of the original groups for the detected gases was performed for all investigated samples as exemplarily summarized for O-MH800-12–125 in Table 2 based on the curves shown in Figure 3.

**Table 2:** Assignment of Peaks in the TPD-EGA Curves (On the Example of Figure 3) According to the Occurrence of Local Maxima or Shoulders at Temperatures (*T*_m_, °C (rounded))^15−18^

*T*_m_	Evolving gas	Assignment	Abbreviation
120	H_2_O	Physisorption	Phys
180	H_2_O	Clusters	Clus
220	CO_2_	Condensed acids	CondAc
240	H_2_O	Condensation	Cond
280	CO	Condensed acids	CondAc
290	CO_2_	Carboxylic acids	CarbAc
430	CO_2_	Anhydrides	Anh
510	CO	Anhydrides	Anh
610	CO_2_	Lactones	Lac
620	CO	Phenols/ethers	PheEth
760	CO	Carbonyls/quinones	CarQui

**Figure 4:**
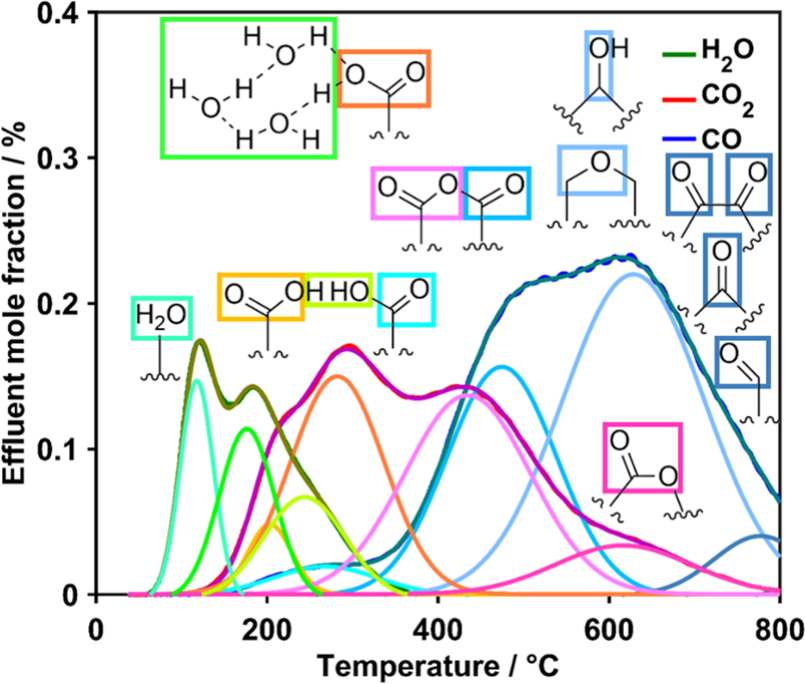
Signal origins of Gaussian contributions obtained by deconvolution of the TPD-EGA spectrum of O-MH800-12-125 with H_2_O contributions in greenish, CO_2_ contributions in reddish, and CO contributions in blueish colors.

Following this general assignment based on local maxima and shoulders observed in the experimental curve, the deconvolution of TPD-EGA was performed, thereby using the restriction for the areas of the anhydride-type OFGs (eq S2) as explained in Section 2.5. The individual contributions derived from the deconvolution are shown in Figure 4 for O-MH800-12-125 and resulted in a modeled curve which is in good agreement with the experimental curve.

The following overall phenomenological conclusions can be drawn: The first contribution to H_2_O release originates from physisorbed molecules. The peak maximum temperature significantly above 100 °C originates from H_2_O adsorbed not only on the outer surface but also inside the pores.^15,21^ Increasing the temperature further, the hydrogen bridges of the clusters formed around carboxylic acids are broken and additional H_2_O is released. Then, the consecutive release of all three detectable gases as the characteristic reaction sequence of two adjacent carboxylic acid groups can be observed. Upon increasing the temperature, these groups condensate resulting in the release of H_2_O and a newly generated anhydride group. This OFG is strongly destabilized and directly releases CO_2_ in a first step. Characteristic for anhydrides in general, a second decomposition step releasing CO follows at slightly higher temperatures. This process occurs overlapping with the decomposition of carboxylic acids resulting in an additional CO_2_ release. Upon further temperature increase, there is the evolution of CO_2_ again directly followed by that of CO typical for anhydrides. These anhydrides were formed already during the functionalization treatment and exhibit a moderate thermal stability. The highest stability of CO_2_-evolving groups is assigned to lactones, whereas further evolution of CO is due to phenols/ethers and carbonyls/quinones as the most stable OFGs.

For all the different extensively functionalized samples, the contributions resulting from the deconvolution of the effluent gas curves recorded during TPD-EGA are shown in Figure S4. The fits for all curves were obtained with a fit quality of *R*^2^ ≥ 0.99 according to eq S3. The corresponding Gaussian fit parameters are summarized in Table S3. The specific amounts of the H_2_O contributions as well as of the individual OFGs were calculated according to eq S4 using the areas derived from the different peaks in the EGA curves. The results are summarized in Table 3, reporting for the differently functionalized samples the total molar amounts of OFGs determined according to eq S4 and the percentage of each specific group relative to the overall population of OFG.

**Table 3:** Total Molar Amounts (*n*_s_, mmol g^–1^) and Percent Contribution of OFGs in the Char Samples Determined According to Equation S4

		Relative concentration of individual OFGs								
Sample	*n* of all OFGs	Phys	Clus	Cond	CondAc	CarbAc	Anh	Lac	PheEth	CarQui
O-MH800-6-125	**9.8**	8.2	5.1	5.1	2.0	15.3	14.3	6.1	39.8	4.1
O-MH800-12-125	**9.9**	5.1	7.1	7.1	2.0	15.2	19.2	5.1	35.4	4.0
O-MH800-18-125	**11.9**	2.5	5.9	7.6	2.5	19.3	17.6	7.6	31.9	5.0
O-MH800-24-125	**14.2**	8.5	8.5	5.6	1.4	17.6	15.5	8.5	28.2	6.3
O-MH800-6-150	**12.2**	3.3	6.6	5.7	1.6	18.0	17.2	7.4	36.1	4.1
O-MH800-6-200	**11.4**	2.6	6.1	6.1	0.9	15.8	17.5	9.6	36.8	4.4

It can be observed that the oxidation treatment under varied conditions resulted in different degrees of functionalization and also in different shares of OFGs. The total amount of evolved gases, i.e., the degree of functionalization, increases progressively with temperature and duration of the treatment, but when the treatment is carried out at 200 °C beyond 6 h, the degree of functionalization starts to decrease. As far as the relative abundance of the different OFGs is concerned, it can be observed that an increase in functionalization temperature relatively favors the generation of anhydrides and lactones, whereas a longer duration resulted in an increased relative occurrence of carboxylic acids and that, overall, more intensified treatments reduce the share of phenols/ethers among OFGs.

### 3.3 Thermicity of OFG Decomposition by TPD–Calorimetric Measurements

**Figure 5:**
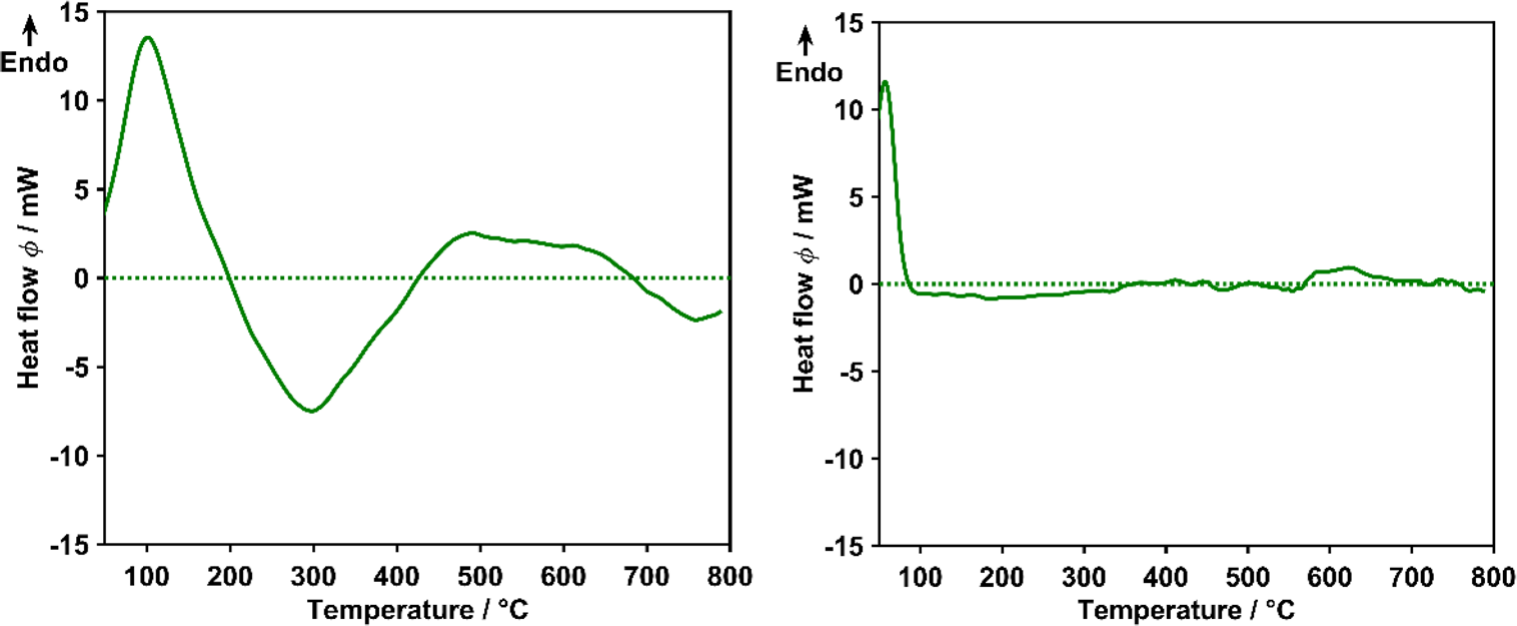
Effect of functionalization on the heat flow curves (left, O-MH800-12-125; right, unfunctionalized MH800).

The typical profile registered for the heat flow curves of functionalized chars is shown in Figure 5(left).

A distinct endothermic peak is observed at temperatures below 200 °C, followed by a pronounced exothermic event in the midtemperature region (200 to 400 °C). More modest thermic effects are recorded above 400 °C, endothermic in the range of 400 to 700 °C and exothermic in the range of 700 to 800 °C. The heat flow curve of the unfunctionalized char shown in Figure 5(right) confirms that changes in the heat flow at temperatures higher than 100 °C are related to the presence of OFGs in the material, whereas thermic effects due to changes in the overall char structure, e.g., caused by thermal annealing, are negligible.

**Figure 6:**
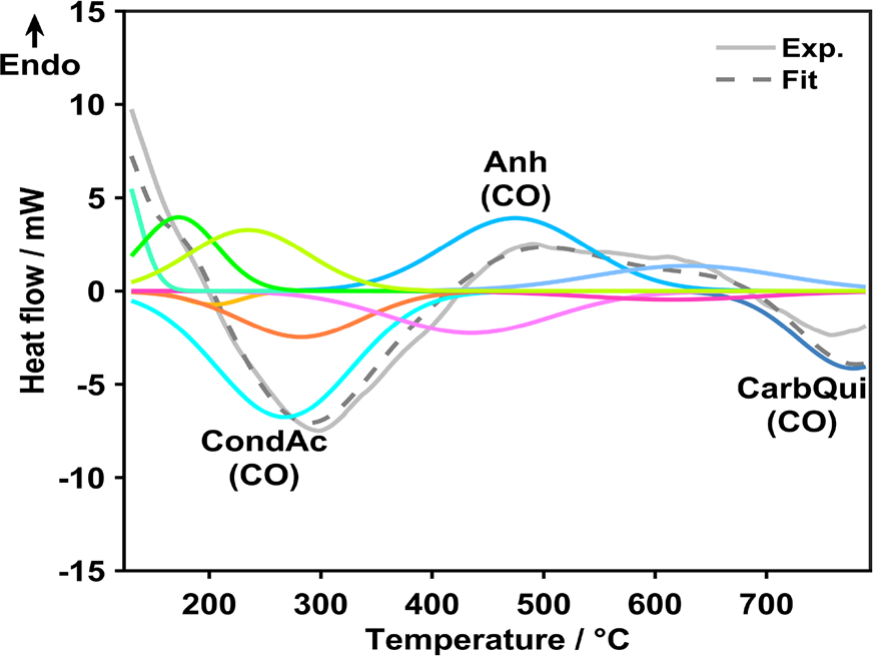
Deconvoluted experimental (Exp.) heat flow curve with Gaussian contributions as assigned directly for the three main contributions as well as in Table 4 for all of the different OFG decompositions, and fitted curve (Fit) for O-MH800-12-125.

The occurrence of exothermic events in the functionalized samples is unexpected, since desorption processes are typically considered endothermic.^25^ However, similar findings have been reported and discussed by Senneca et al.^22^ for coal. The exothermic effects observed for the functionalized chars suggest that upon decomposition of OFGs more than solely desorption of carbon oxides occurs. In order to investigate the origin of the observed effects, a clear assignment to the individual OFGs is required, which can be obtained using the deconvolution results of the heat flow curve shown in Figure 6 (Table 4) for O-MH800-12-125.

**Table 4:**
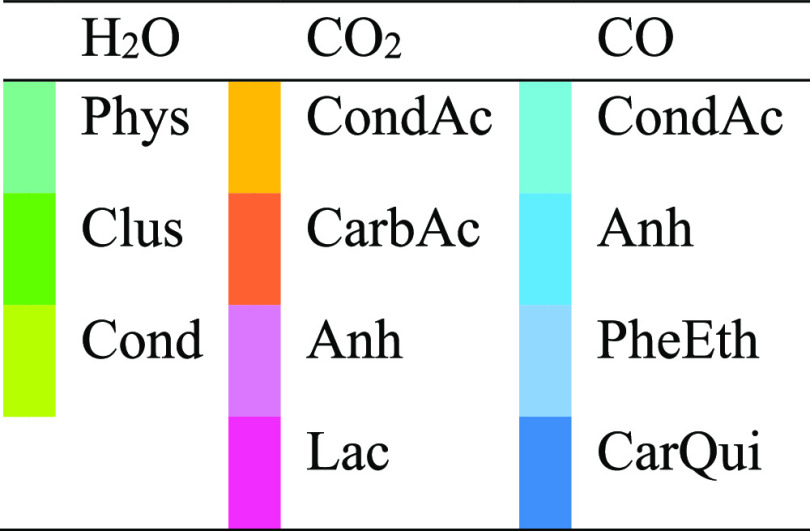
Assignment of Fit Contributions in the Heat Flow Curve (Figure 6) for the Different Evolving Gases with Increasing Thermal Stability from Top to Bottom

Similar results were obtained with all of the extensively functionalized samples (Figure S5). At the lower end of the temperature range, the amount of released H_2_O was not well reproducible and shown to be not solely correlated to functionalization. Therefore, the reliable correlation of heat flow to evolving gases was restricted to *T* ≥ 130 °C, and the deconvolution procedure of the heat flow signals was performed only in this temperature range. When comparing experimental and fitted heat flow curves, an overall good agreement was found with slight deviations mostly in the higher temperature range. These differences may be caused by experimental and/or modeling uncertainties, since 800 °C is close to the upper temperature limit of the calorimeter of 830 °C. Furthermore, it was not possible to distinguish phenols/ethers as well as carbonyls/quinones. Therefore, different relative amounts of these species in the samples may generate discrepancies between the experiment and the fit in the high-temperature region.

The specific thermicities derived for the individual OFG contributions to the heat flow are summarized in Table 5.

**Table 5:** Molar Thermicity (*Q*_m_, kJ mol^–1^; Equation S6) and Evolving Gases of Assigned Signal Origins

Signal origin	Evolving gas	*Q*_m_
Physisorption	H_2_O	135
Clusters	H_2_O	103
Condensation	H_2_O	121
Condensed acids	CO_2_	–41
Carboxylic acids	CO_2_	–44
Anhydrides	CO_2_	–44
Lactones	CO_2_	–36
Condensed acids	CO	–920
Anhydrides	CO	68
Phenols/ethers	CO	16
Carbonyls/quinones	CO	–296

As expected, the H_2_O release was endothermic in each case (*Q*_m_ > 100 kJ mol^–1^), whereas the CO_2_ release of all of the different originating OFGs was exothermic by about −40 kJ mol^–1^. The evolution of CO from condensed acids and carbonyls/quinones turned out to be strongly exothermic. Remarkably, the overall heat effect of −31 kJ g^–1^ C reported for CO_*x*_ desorption from oxidized coal^22^ is equal to a molar heat effect of −372 kJ mol^–1^. As this heat has been detected concurrent to CO_*x*_ evolution mainly above 650 °C, carbonyls/quinones are likely the originating groups. Thus, the molar thermicity of −296 kJ mol^–1^ derived for the decomposition of carbonyls/quinones in this work is well in line with the previously reported exothermic effect despite the differences in char material and measurement procedure. Differently, the evolution of CO from anhydrides and phenols/ethers was derived to be endothermic. In fact, the decomposition of anhydrides composed of an exothermic CO_2_ release and an endothermic CO release is altogether moderately endothermic (24 kJ mol^–1^). It must also be noted that the thermicity of CO_2_ release from anhydrides directly generated during the functionalization treatment and from those generated during the TPD by condensation of adjacent acid groups is comparable, but the thermicity assigned to the subsequent release of CO differs strongly. In the case of anhydrides generated by acid condensation, a pronounced exothermic effect was recorded so that the overall decomposition (CO + CO_2_) of anhydrides generated during TPD is in total highly exothermic (−961 kJ mol^–1^).

### 3.4 Proposed Reaction Scheme and Theoretical Calculations

**Figure 7:**
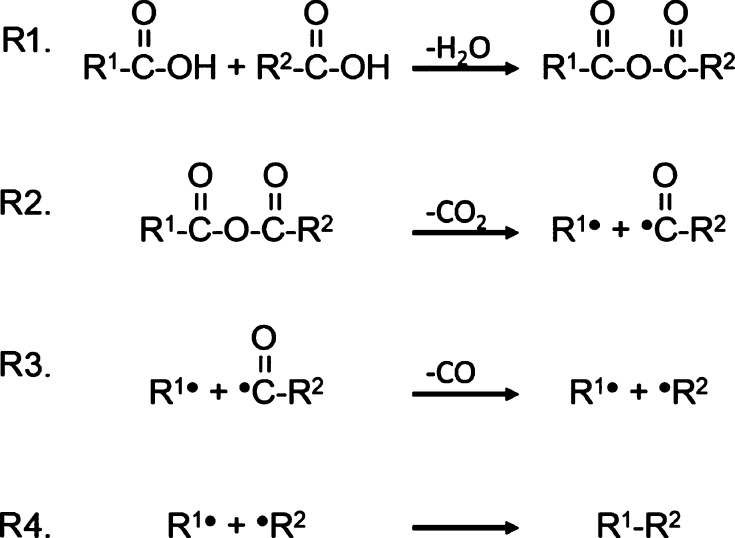
Scheme of reactions of adjacent carboxylic acids.

The remarkable thermicity associated with the decomposition of condensed acids suggests that carbon matrix rearrangements are the dominating processes rather than the desorption of the released oxides.^22,25^ Taking also the preceding step of condensation into account, the reaction scheme shown in Figure 7 is proposed based on the results of the heat flow measurements.

The heat of reaction for steps R1 and R2 is equal to the experimentally measured heats of condensation (121 kJ mol^–1^) and decarboxylation (−41 kJ mol^–1^), whereas the combined heat of decarbonylation and recombination (steps R3 and R4) is equal to the heat measured upon CO evolution (−920 kJ mol^–1^).

For a closer investigation of the proposed reaction scheme, theoretical calculations by means of DFT and MD simulations employing (xTB) were performed. Different char models have been developed with different OFGs in order to focus on relative thermal stabilities. Details on DFT calculations and on char models are provided in the Supporting Information (Section S5). The first model contained no OFGs besides the pair of adjacent acid groups to be studied (Figure S6). In the second model, the degree of functionalization was increased by the incorporation of additional OFGs, while in the third model two carbon layers were used (two-sheet model). Furthermore, larger char models were created to be used in an MD simulation. As these simulations refer to a rather simplified char structure models, they are not expected to reproduce the experimental results quantitatively. However, they allow one to disclose the mechanisms of reaction and the effect of characteristic char properties. Table 6 summarizes the reaction energies found for the individual reaction steps shown in Figure 7 for models of different complexity.

**Table 6:** Reaction Energies (kJ mol^–1^) Calculated on the TPSS/def2-SVP Level of Theory for the Reactions Depicted in Figure 7 Using Different Char Models

Step	Minimal OFG	Additional OFGs	Two sheets
R1	99.60	72.54	62.79
R2	53.21	–1.56	20.93
R3 + R4	238.22	439.56	124.80

For the minimalistic model containing only the adjacent carboxylic acids, the calculations predict strongly endothermic effects which do not match the observed exothermicities for step R2 and steps R3 + R4. Considering a more complex model with an increased concentration of OFGs in proximity of the reacting OFGs, the calculations predict also an exothermic effect associated with the decarboxylation (step R2). Although for all models an endothermic effect is predicted for steps R3 + R4, the implementation of an additional char layer was found to largely decrease this endothermicity. Therefore, the experimentally determined large exothermicity assigned to steps R3 + R4 is likely to originate from rearrangements in the char structure involving several carbon layers. This is backed up by the comparison of the minimal model with the two-sheet model. In a future study one could increase the number of sheets in the model for the DFT calculation, but the computational time quickly increases due to the cubic to quartic scaling of DFT calculations. Furthermore, we want to note in passing that in extended aromatic systems even local reactions are affected by long-range effects of the aromatic system. This is a further point which could explain the discrepancy.

In order to investigate larger systems to gain further insights, an MD simulation of the dynamics of OFG decomposition was performed with larger char models (see Supporting Information Section S5). Although this simulation was run at rather high temperatures (727 to 1727 °C) to trigger a fast decomposition, the overall system remained relatively stable for the span of the simulation time. Nevertheless, the simulation was able to confirm the experimentally observed comparatively low temperature stability of anhydrides.

## 4 Conclusions

The thermicity associated with the decomposition of different OFGs on the surface of HNO_3_ vapor-functionalized mineral-free synthetic carbon derived from cellulose was investigated experimentally as well as theoretically. TPD experiments with gas and calorimetric measurements up to 800 °C allowed one to assess the absolute amounts of individual OFGs present on the different samples upon oxidation treatments and their contributions to the heat flow. Three main conclusions regarding the thermicity of OFG decomposition can be drawn: The decomposition of phenols/ethers is endothermic. The decomposition of carboxylic acids, lactones, and carbonyls/quinones is exothermic. The two-step decomposition of anhydrides is composed of an exothermic and a slightly stronger endothermic contribution in the case of anhydrides directly present on the chars, whereas it is strongly exothermic in the case of anhydrides generated by acid condensation during the TPD measurements.

The proposed reaction scheme for adjacent acids is composed of condensation, decarboxylation, and decarbonylation as well as recombination. Theoretical calculations suggest the moderate exothermicity of decarboxylation to be induced by the presence of neighboring OFGs, whereas the strong exothermic effect measured for decarbonylation and recombination likely originates from accompanying structural rearrangements in the char matrix involving several carbon layers.

## Supporting Information Available

Samples functionalized by molecular oxygen, types of OFGs and the detailed experimental procedure of their characterization by XPS, TPD-EGA, and calorimetric TPD measurements including the deconvolution procedure of obtained TPD curves, calorimetric TPD curves of the less functionalized samples, additional deconvolution data from the TPD-EGA and calorimetric TPD measurements, and figures of the theoretical models (PDF)

## References

[ref1] García-García F. R.; Gallegos-Suarez E.; Fernández-García M.; Guerrero-Ruiz A.; Rodríguez-Ramos I. Understanding the role of oxygen surface groups: The key for a smart ruthenium-based carbon-supported heterogeneous catalyst design and synthesis. Appl. Catal., A 2017, 544, 66–76. 10.1016/j.apcata.2017.06.030.

[ref2] Gosselink R.W.; van den Berg R.; Xia W.; Muhler M.; de Jong K.P.; Bitter J.H. Gas phase oxidation as a tool to introduce oxygen containing groups on metal-loaded carbon nanofibers. Carbon 2012, 50, 4424–4431. 10.1016/j.carbon.2012.05.020.

[ref3] Reiche S.; Kowalew N.; Schlögl R. Influence of synthesis pH and oxidative strength of the catalyzing acid on the morphology and chemical structure of hydrothermal carbon. ChemPhysChem 2015, 16, 579–587. 10.1002/cphc.201402834. 25511815

[ref4] Otake Y.; Jenkins R. G. Examination of oxygen functional groups on carbonaceous solids by linear temperature desorption techniques. Prepr. Pap., Am. Chem. Soc., Div. Fuel Chem. 1987, 32, 310–317.

[ref5] Boehm H. Surface oxides on carbon and their analysis: a critical assessment. Carbon 2002, 40, 145–149. 10.1016/S0008-6223(01)00165-8.

[ref6] Boehm H. P. Some aspects of the surface chemistry of carbon blacks and other carbons. Carbon 1994, 32, 759–769. 10.1016/0008-6223(94)90031-0.

[ref7] Kundu S.; Wang Y.; Xia W.; Muhler M. Thermal Stability and Reducibility of Oxygen-Containing Functional Groups on Multiwalled Carbon Nanotube Surfaces: A Quantitative High-Resolution XPS and TPD/TPR Study. J. Phys. Chem. C 2008, 112, 16869–16878. 10.1021/jp804413a.

[ref8] Dandekar A.; Baker R.; Vannice M. A. Characterization of activated carbon, graphitized carbon fibers and synthetic diamond powder using TPD and DRIFTS. Carbon 1998, 36, 1821–1831. 10.1016/S0008-6223(98)00154-7.

[ref9] Rocha R. P.; Silva A. M. T.; Romero S. M. M.; Pereira M. F. R.; Figueiredo J. L. Figueiredo. The role of O- and S-containing surface groups on carbon nanotubes for the elimination of organic pollutants by catalytic wet air oxidation. Appl. Catal., B 2014, 147, 314–321. 10.1016/j.apcatb.2013.09.009.

[ref10] Wen G.; Wang B.; Wang C.; Wang J.; Tian Z.; Schlögl R.; Su D. S. Hydrothermal Carbon Enriched with Oxygenated Groups from Biomass Glucose as an Efficient Carbocatalyst. Angew. Chem., Int. Ed. Engl. 2017, 56, 600–604. 10.1002/anie.201609047. 27925400

[ref11] Chen C.-M.; Zhang Q.; Yang M.-G.; Huang C.-H.; Yang Y.-G.; Wang M.-Z. Structural evolution during annealing of thermally reduced graphene nanosheets for application in supercapacitors. Carbon 2012, 50, 3572–3584. 10.1016/j.carbon.2012.03.029.

[ref12] Karlström O.; Brink A.; Hupa M. Desorption kinetics of CO in char oxidation and gasification in O2, CO2 and H2O. Combust. Flame 2015, 162, 788–796. 10.1016/j.combustflame.2014.08.010.

[ref13] Montoya A.; Mondragón F.; Truong T. N. Formation of CO precursors during char gasification with O2, CO2 and H2O. Fuel Process. Technol. 2002, 77–78, 125–130. 10.1016/S0378-3820(02)00013-9.

[ref14] Haynes B. S. A turnover model for carbon reactivity I. development. Combust. Flame 2001, 126, 1421–1432. 10.1016/S0010-2180(01)00263-2.

[ref15] Düngen P.; Schlögl R.; Heumann S. Non-linear thermogravimetric mass spectrometry of carbon materials providing direct speciation separation of oxygen functional groups. Carbon 2018, 130, 614–622. 10.1016/j.carbon.2018.01.047.

[ref16] Friedel Ortega K.; Arrigo R.; Frank B.; Schlögl R.; Trunschke A. Acid-Base Properties of N-Doped Carbon Nanotubes: A Combined Temperature-Programmed Desorption, X-ray Photoelectron Spectroscopy, and 2-Propanol Reaction Investigation. Chem. Mater. 2016, 28, 6826–6839. 10.1021/acs.chemmater.6b01594.

[ref17] Godino-Salido M. L.; López-Garzón R.; Gutiérrez-Valero M. D.; Arranz-Mascarós P.; Melguizo-Guijarro M.; López de la Torre M. D.; Gómez-Serrano V.; Alexandre-Franco M.; Lozano-Castelló D.; Cazorla-Amorós D.; Domingo-García M. Effect of the surface chemical groups of activated carbons on their surface adsorptivity to aromatic adsorbates based on π-π interactions. Mater. Chem. Phys. 2014, 143, 1489–1499. 10.1016/j.matchemphys.2013.12.005.

[ref18] Tamargo-Martínez K.; Villar-Rodil S.; Martínez-Alonso A.; Tascón J. Chemical and structural modifications of carbon nanofibers with different degrees of graphitic order following oxygen plasma treatments. Mater. Chem. Phys. 2013, 138, 615–622. 10.1016/j.matchemphys.2012.12.028.

[ref19] Figueiredo J. L.; Pereira M. F. R. The role of surface chemistry in catalysis with carbons. Catal. Today 2010, 150, 2–7. 10.1016/j.cattod.2009.04.010.

[ref20] Li N.; Ma X.; Zha Q.; Kim K.; Chen Y.; Song C. Maximizing the number of oxygen-containing functional groups on activated carbon by using ammonium persulfate and improving the temperature-programmed desorption characterization of carbon surface chemistry. Carbon 2011, 49, 5002–5013. 10.1016/j.carbon.2011.07.015.

[ref21] Rosenthal D.; Ruta M.; Schlögl R.; Kiwi-Minsker L. Combined XPS and TPD study of oxygen-functionalized carbon nanofibers grown on sintered metal fibers. Carbon 2010, 48, 1835–1843. 10.1016/j.carbon.2010.01.029.

[ref22] Senneca O.; Salatino P.; Cortese L. Assessment of the thermochemistry of oxygen chemisorption and surface oxide desorption during looping combustion of coal char. Proc. Combust. Inst. 2013, 34, 2787–2793. 10.1016/j.proci.2012.07.051.

[ref23] Zou R.; Luo G.; Cao L.; Jiang L.; Li X.; Yao H. Surface CO/CO2 ratio of char combustion measured by thermogravimetry and differential scanning calorimetry. Fuel 2018, 233, 480–485. 10.1016/j.fuel.2018.06.072.

[ref24] Cerciello F.; Senneca O.; Coppola A.; Forgione A.; Lacovig P.; Salatino P. The influence of temperature on the nature and stability of surface-oxides formed by oxidation of char. Renewable Sustainable Energy Rev. 2021, 137, 110595 10.1016/j.rser.2020.110595.

[ref25] Levi G.; Causà M.; Lacovig P.; Salatino P.; Senneca O. Mechanism and Thermochemistry of Coal Char Oxidation and Desorption of Surface Oxides. Energy Fuels 2017, 31, 2308–2316. 10.1021/acs.energyfuels.6b02324.

[ref26] Liu G.-S.; Niksa S. Coal conversion submodels for design applications at elevated pressures. Part II. Char gasification. Prog. Energy Combust. Sci. 2004, 30, 679–717. 10.1016/j.pecs.2004.08.001.

[ref27] Hurt R. H.; Calo J. M. Semi-global intrinsic kinetics for char combustion modeling. Combust. Flame 2001, 125, 1138–1149. 10.1016/S0010-2180(01)00234-6.

[ref28] Jimenez-Orozco C.; Mondragón F.; Espinal J. F. Experimental and Computational Analysis of the Formation of Surface Oxygen Functional Groups during Iron Catalyzed Char Gasification with CO2. Combust. Sci. Technol. 2018, 190, 687–706. 10.1080/00102202.2017.1405948.

[ref29] Xu K.; Ye P. D. Theoretical Study on the Oxidation Mechanism and Dynamics of the Zigzag Graphene Nanoribbon Edge by Oxygen and Ozone. J. Phys. Chem. C 2014, 118, 10400–10407. 10.1021/jp500633w.

[ref30] Wang Z.; Yang B.; Wang Y.; Zhao Y.; Cao X.-M.; Hu P. Identifying the trend of reactivity for sp2 materials: an electron delocalization model from first principles calculations. Phys. Chem. Chem. Phys. 2013, 15, 9498–9502. 10.1039/c3cp51375k. 23685875

[ref31] Sendt K.; Haynes B. S. Density functional study of the reaction of O2 with a single site on the zigzag edge of graphene. Proc. Combust. Inst. 2011, 33, 1851–1858. 10.1016/j.proci.2010.06.021.

[ref32] Radovic L. R. Active sites in graphene and the mechanism of CO2 formation in carbon oxidation. J. Am. Chem. Soc. 2009, 131, 17166–17175. 10.1021/ja904731q. 19891428

[ref33] Huang Y.; Cannon F. S.; Watson J. K.; Reznik B.; Mathews J. P. Activated carbon efficient atomistic model construction that depicts experimentally-determined characteristics. Carbon 2015, 83, 1–14. 10.1016/j.carbon.2014.11.012.

[ref34] Roberts M. J.; Everson R. C.; Domazetis G.; Neomagus H. W.; Jones J. M.; van Sittert C. G.; Okolo G. N.; van Niekerk D.; Mathews J. P. Density functional theory molecular modelling and experimental particle kinetics for CO2-char gasification. Carbon 2015, 93, 295–314. 10.1016/j.carbon.2015.05.053.

[ref35] Wang C.; Watson J. K.; Louw E.; Mathews J. P. Construction Strategy for Atomistic Models of Coal Chars Capturing Stacking Diversity and Pore Size Distribution. Energy Fuels 2015, 29, 4814–4826. 10.1021/acs.energyfuels.5b00816.

[ref36] Pflieger C.; Lotz K.; Hilse N.; Berger C. M.; Schiemann M.; Debiagi P.; Hasse C.; Scherer V.; Muhler M. Catalytic influence of mineral compounds on the reactivity of cellulose-derived char in O2-, CO2-, and H2O-containing atmospheres. Fuel 2021, 287, 119584 10.1016/j.fuel.2020.119584.

[ref37] Balasubramani S. G.; Chen G. P.; Coriani S.; Diedenhofen M.; Frank M. S.; Franzke Y. J.; Furche F.; Grotjahn R.; Harding M. E.; Hättig C.; Hellweg A.; Helmich-Paris B.; Holzer C.; Huniar U.; Kaupp M.; Marefat Khah A.; Karbalaei Khani S.; Müller T.; Mack F.; Nguyen B. D.; Parker S. M.; Perlt E.; Rappoport D.; Reiter K.; Roy S.; Rückert M.; Schmitz G.; Sierka M.; Tapavicza E.; Tew D. P.; van Wüllen C.; Voora V. K.; Weigend F.; Wodyński A.; Yu J. M. TURBOMOLE: Modular program suite for ab initio quantum-chemical and condensed-matter simulations. J. Chem. Phys. 2020, 152, 184107 10.1063/5.0004635. 32414256 PMC7228783

[ref38] TURBOMOLE, v7.5; TURBOMOLE GmbH 2020. https://www.turbomole.org/ (accessed 2022-05-22).

[ref39] Xia W.; Jin C.; Kundu S.; Muhler M. A highly efficient gas-phase route for the oxygen functionalization of carbon nanotubes based on nitric acid vapor. Carbon 2009, 47, 919–922. 10.1016/j.carbon.2008.12.026.

[ref40] Figueiredo J. L.; Pereira M. F. R.; Freitas M. M. A.; Órfão J. J. M. Characterization of Active Sites on Carbon Catalysts. Ind. Eng. Chem. Res. 2007, 46, 4110–4115. 10.1021/ie061071v.

[ref41] Levi G.; Senneca O.; Causà M.; Salatino P.; Lacovig P.; Lizzit S. Probing the chemical nature of surface oxides during coal char oxidation by high-resolution XPS. Carbon 2015, 90, 181–196. 10.1016/j.carbon.2015.04.003.

[ref42] Ishii T.; Kashihara S.; Hoshikawa Y.; Ozaki J.; Kannari N.; Takai K.; Enoki T.; Kyotani T. A quantitative analysis of carbon edge sites and an estimation of graphene sheet size in high-temperature treated, non-porous carbons. Carbon 2014, 80, 135–145. 10.1016/j.carbon.2014.08.048.

[ref43] Figueiredo J.; Pereira M.; Freitas M.; Órfão J. Modification of the surface chemistry of activated carbons. Carbon 1999, 37, 1379–1389. 10.1016/S0008-6223(98)00333-9.

[ref44] Likodimos V.; Steriotis T. A.; Papageorgiou S. K.; Romanos G. E.; Marques R. R.; Rocha R. P.; Faria J. L.; Pereira M. F.; Figueiredo J. L.; Silva A. M.; Falaras P. Controlled surface functionalization of multiwall carbon nanotubes by HNO_3_ hydrothermal oxidation. Carbon 2014, 69, 311–326. 10.1016/j.carbon.2013.12.030.

[ref45] Toebes M. L.; van Heeswijk J. M.; Bitter J. H.; Jos van Dillen A.; de Jong K. P. The influence of oxidation on the texture and the number of oxygen-containing surface groups of carbon nanofibers. Carbon 2004, 42, 307–315. 10.1016/j.carbon.2003.10.036.

[ref46] Do D. D.; Do H. D. A model for water adsorption in activated carbon. Carbon 2000, 38, 767–773. 10.1016/S0008-6223(99)00159-1.

