# Supplementary material for: Thermicity of the Decomposition of Oxygen Functional Groups on Cellulose-Derived Chars
Source: ACS Omega. 2022 Dec 15;7(51):48606–14. doi: 10.1021/acsomega.2c07429 (PMC9798768; doi:10.1021/acsomega.2c07429)
Supplement: Supplementary file 1 — ao2c07429_si_001.pdf [file ao2c07429_si_001.pdf]

Thermicity of the Decomposition of Oxygen Functional Groups on Cellulose-Derived Chars

-

Supplementary Information

Christin Pflieger,[†] *Till Eckhard,*[†] *Gunnar Schmitz,*[‡] *Vanessa Angenent,*[‡]

Maximilian Göckeler,[†] *Osvalda Senneca,*[¶] *Rochus Schmid,*[‡] *Francesca Cerciello,*[*,†]

and Martin Muhler[*,†]

[†]Laboratory of Industrial Chemistry, Ruhr University Bochum, 44801 Bochum, Germany

[‡]Computational Materials Chemistry Group, Ruhr University Bochum, 44801 Bochum, Germany

[¶]Istituto di Scienze e Tecnologie per l'Energia e la Mobilità Sostenibili, Consiglio Nazionale delle Ricerche, 80125 Napoli, Italy

S1 Supplementary Information on Char Oxidation by Molecular Oxygen

S1.1 Functionalization by Molecular Oxygen

In addition to the samples functionalization using HNO_3 vapor, char oxidation by molecular oxygen was also performed. MH800 was placed on a frit in a vertical quartz reactor (inner diameter 8 mm, outer diameter 10 mm, length 250 mm) inside a split tube furnace (LK Platin 1350-17-100-1 by HTM Reetz) of a flow setup. The sample temperature was measured by a type K Inconel600 coated thermocouple, while the effluent gas detection of CO/CO_2 and O_2 was performed by a multi-channel analyzer (NGA 2000 MLT4 by Rosemount) as well as a paramagnetical detector (Magnos 16 by Hartmann & Braun) downstream of the reactor. Oxidation was carried out at 200 °C and 425 °C. For the experiment at 200 °C, the sample was heated with 10 °Cmin^{-1} in Ar (99.999% purity), and then the flow was immediately switched to an oxidative atmosphere of 20 mLmin^{-1} 20% O_2/Ar (20.00(40)% actual composition). The sample was kept under these conditions for 6 h and then cooled in Ar. During oxidation it was checked that CO_x evolution was negligible and no significant char conversion occurred. Differently, at 425 °C the isothermal oxidation lasted about 0.5 h which corresponded to a mass loss of 10%. Further conversion was undesirable as the differences in the carbon structure would complicate any comparison to other char samples. The char samples oxidized by molecular oxygen were labelled as 'O-MH800-O_2-T_{ox}' where T_{ox} is the temperature of the oxidation experiment. For example, MH800 oxidized by molecular oxygen at 200 °C is denoted as 'O-MH800-O_2-200'.

S1.2 Characterization of Samples Oxidized by Molecular Oxygen

Table S1. Elemental compositions w in wt% of the O_2-functionalised char samples (sulfur- and mineral-free, N may result from pyrolysis in N_2) in comparison to the char prior to functionalization.

Sample	w_C	w_H	w_N	w_O[a]
MH800	95.5	0.8	0.0	3.7
O-MH800-O_2-200	89.8	1.2	0.3	8.6
O-MH800-O_2-425	77.5	2.0	0.0	20.4

[a]Calculated as difference (Eq. (1)).

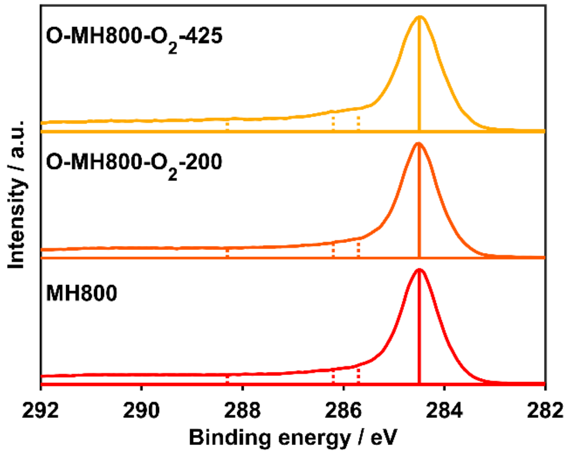

Figure S1. Normalized XPS spectra (C 1s region) of the O_2-functionalised char samples in comparison to the char prior to functionalization, with solid vertical lines indicating visible peaks and the dashed lines indicating further expected peak positions.

S2 Supplementary Information on Char Characterization Procedures

S2.1 OFGs present on the Char Surface

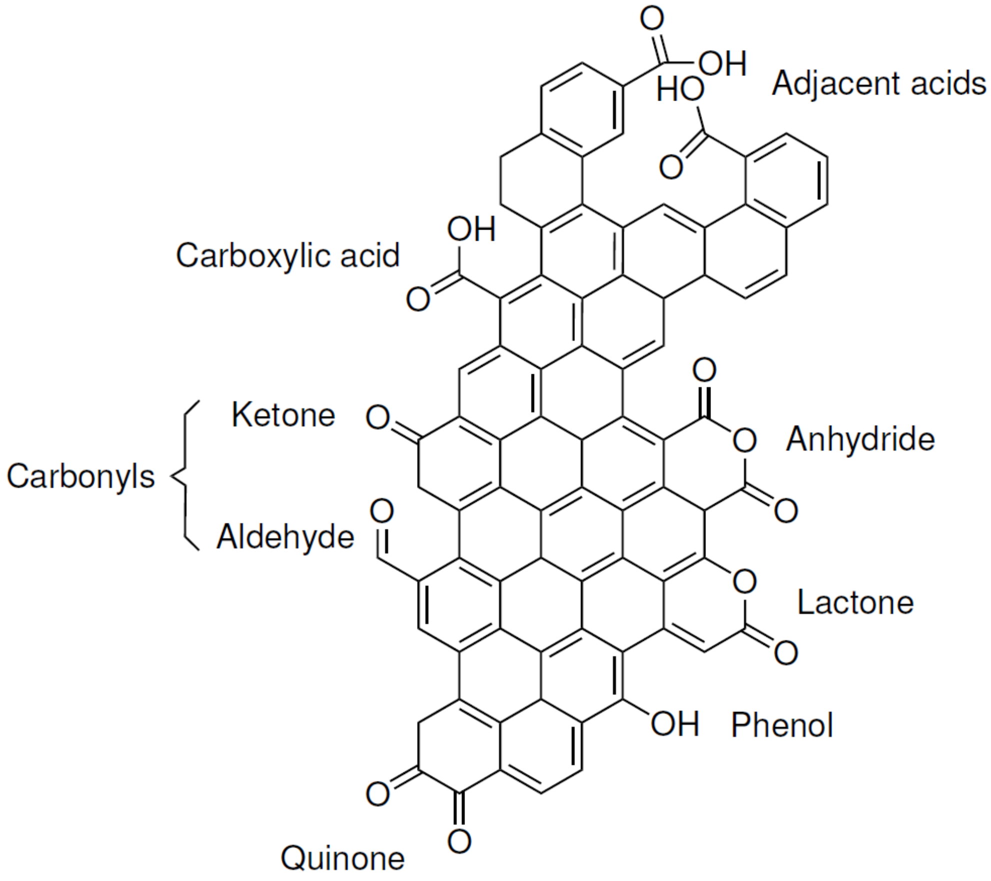

Figure S2. Exemplary OFGs formed on carbon surfaces (based on Gosselink *et al.*).[1]

S2.2 XPS measurements

XPS measurements for the characterization of OFGs were performed in a ultrahigh vacuum setup which was equipped with a mono-chromatic Al Kα X-ray source (anode operated at 14.5 kV and 30 mA; 1486.6 eV) and a high-resolution Gammadata-Scienta SES 2002 hemispherical analyzer. During the measurements, a base pressure of about 1×10^{-9} mbar was maintained in the measurement chamber and a flood gun was used to compensate charging effects. For both energy regions, the spectra were recorded in fixed transmission mode with a pass energy of 200 eV and measurements were performed five times in succession with six to ten iterations. Finally,

calibration on the graphitic carbon peak at 284.5 eV and normalization to its maximum intensity were performed.

S2.3 TPD Measurements with an Extensive Evolved Gas Analysis

TPD measurements with an extensive evolved gas analysis (EGA) were carried out in a two-part quartz reactor (inner diameter 7mm/3mm, length 370mm + 330mm) ensuring a defined sample position. In each experiment 60 mg of char sample were placed between two quartz wool plugs. The reactor was placed inside a horizontal three-zone tube furnace (TZF 12/38/400 by Carbolite). The samples were heated with 5 °Cmin^{-1} to 800 °C in a constant flow of 50 mL min^{-1} N_2 (99.999% purity). The sample temperature was detected by a type N thermocouple (± 1 °C) and downstream, the effluent gases were analyzed by a multi-channel analyzer (NGA 2000 MLT4 by Rosemount) which enabled the quantification of H_2O, CO_2, and CO in a range of 0% to 1%.

S2.4 Calorimetric TPD Measurements

Calorimetric TPD measurements were carried out analogous to the TPD-EGA measurements. In each experiment 60 mg of char sample were placed on a frit in a quartz reactor (inner diameter 3mm, length 200mm). The reactor was placed inside the vertical sample cell of a Tian-Calvet type calorimeter (SENSYS DSC by Setaram) implemented in a flow-setup. The calorimeter was calibrated by the Joule-Thompson effect and regularly checked using phase transformation reactions of inorganic compounds. The calorimeter was heated with 5 °Cmin^{-1} to 800 °C. A flow of 50 mL min^{-1} He (99.999% purity) was equally split through the reactor containing the char sample and the reference cell. The heat flow was detected as a function of temperature (± 1 °C). A strong baseline drift was observed throughout the large temperature range due to the high amount

of sample. In order to isolate the OFG-related thermic effects, each sample measurement was followed by an analogous blank measurement of this same char sample with already decomposed OFGs.[2] A multi-channel detector (NGA 2000 MLT4 by Rosemount) downstream of the calorimeter was used to monitor the evolving CO_x in order to check the concordance of the calorimetric TPD measurements with the TPD-EGA measurement. Notably, this allowed also to check the absence of any evolving gases during the blank measurement.

S2.5 Deconvolution of Effluent Gas and Heat Flow Curves

The H_2O, CO_2, and CO release curves throughout TPD-EGA were deconvoluted assuming Gaussian distributions based on previous literature.[39] The assignment of EGA peaks to different OFGs is discussed in Section 3.2. The parameters obtained by the deconvolution of the EGA curves were then used to deconvolute the heat flow curves of the calorimetric TPD experiments, in which the contributions of different OFGs were not individually resolved. The deconvolution of detected effluent fractions as a function of temperature was carried out considering the Gaussian contributions of all assigned functional groups j to each of the evolved gas species curves i:

$$x_i(T) = x_{0,j} + \sum_j x_j = x_{0,j} + \sum_j \frac{A_j}{v_j \cdot \sqrt{\frac{\pi}{2}}} \cdot exp\left(\frac{-2(T-T_{c,j})^2}{v_j^2}\right) \quad \text{(S1)}$$

with a general offset x_0 as well as area A, full width at half maximum v, and centre temperature T_C as Gaussian parameters. However, as anhydrides are known to decompose into CO and CO_2 of equal amounts, the deconvolution was further restricted by specifying equal areas for condensed acids (CondAc) and anhydrides (Anh):[3,4]

$$A_{CondAc,CO} = A_{CondAc,CO_2} \text{ and } A_{Anh,CO} = A_{Anh,CO_2} \quad \text{(S2)}$$

The effluent curves of the individual char samples were deconvoluted by least-square fitting based on the interior-point algorithm in MATLAB R2020b with 1×10^6 as the maximum number of iterations, complying to a tolerance of 1×10^{-10}. The fit quality R^2 relating experimental values (Exp.) to fitted values (Fit) was derived for measurement points z of a curve J as

$$R^2 = 1 - \frac{\sum_z \left(J_{\text{Exp.}}(z) - J_{\text{Fit}}(z)\right)^2}{\sum_z \left(J_{\text{Exp.}}(z)\right)^2} \quad \text{(S3)}$$

Using the area parameters of the different Gaussian contributions, the specific molar amounts n_s of assigned OFGs were calculated using the ideal gas law including temperature T_{cc} and pressure p_{cc} as mass flow controller calibration conditions, the universal gas constant R, and the adjusted volume flow of inert gas $\dot{V}$ as well as the used sample mass m_S:

$$n_{s,j} = \frac{p_{cc} \cdot \dot{V} \cdot A_j}{R \cdot T_{cc} \cdot m_S \cdot 100} \quad \text{(S4)}$$

While the effluent gas curves were deconvoluted for the different samples separately, an optimization of one set of thermicity fit parameters was performed for the heat flow curves of all samples concurrently. This deconvolution was based on the assumption that thermic effects assigned to OFG decomposition occurred concurrently with the evolution of decomposition products resulting in Gaussian contributions of OFGs at the same center temperature and of the same width in the heat flow curve and the effluent gas curves. Accordingly, the peak areas of the heat flow curve contributions ϕ_j were fitted proportional to those of the absolute molar amounts of effluent gases n, derived from the product of specific molar amounts n_s and used sample mass m_s:

$$n = n_s \cdot m_s \quad \text{(S5)}$$

The molar thermicity Q_m is a proportionality factor specific for the different assigned OFGs but constant throughout the sample series:

$$\phi_j = Q_{m,j} \cdot \frac{n_j}{v_j \cdot \sqrt{\frac{\pi}{2}}} \cdot exp\left(\frac{-2\left(T-T_{c,j}\right)^2}{v_j^2}\right) \tag{S6}$$

This proportionality was possible due to similar reaction conditions in both TPD-EGA and calorimetric TPD measurements. Further, the deconvolution procedure was based on the assumption that thermicity is sufficiently determined by focusing on the decomposing OFG, whereas the influence of neighboring OFGs or slight changes in the overall char structure due to the increased temperature such as thermal annealing were of negligible influence.

S3 Supplementary Calorimetric TPD measurements

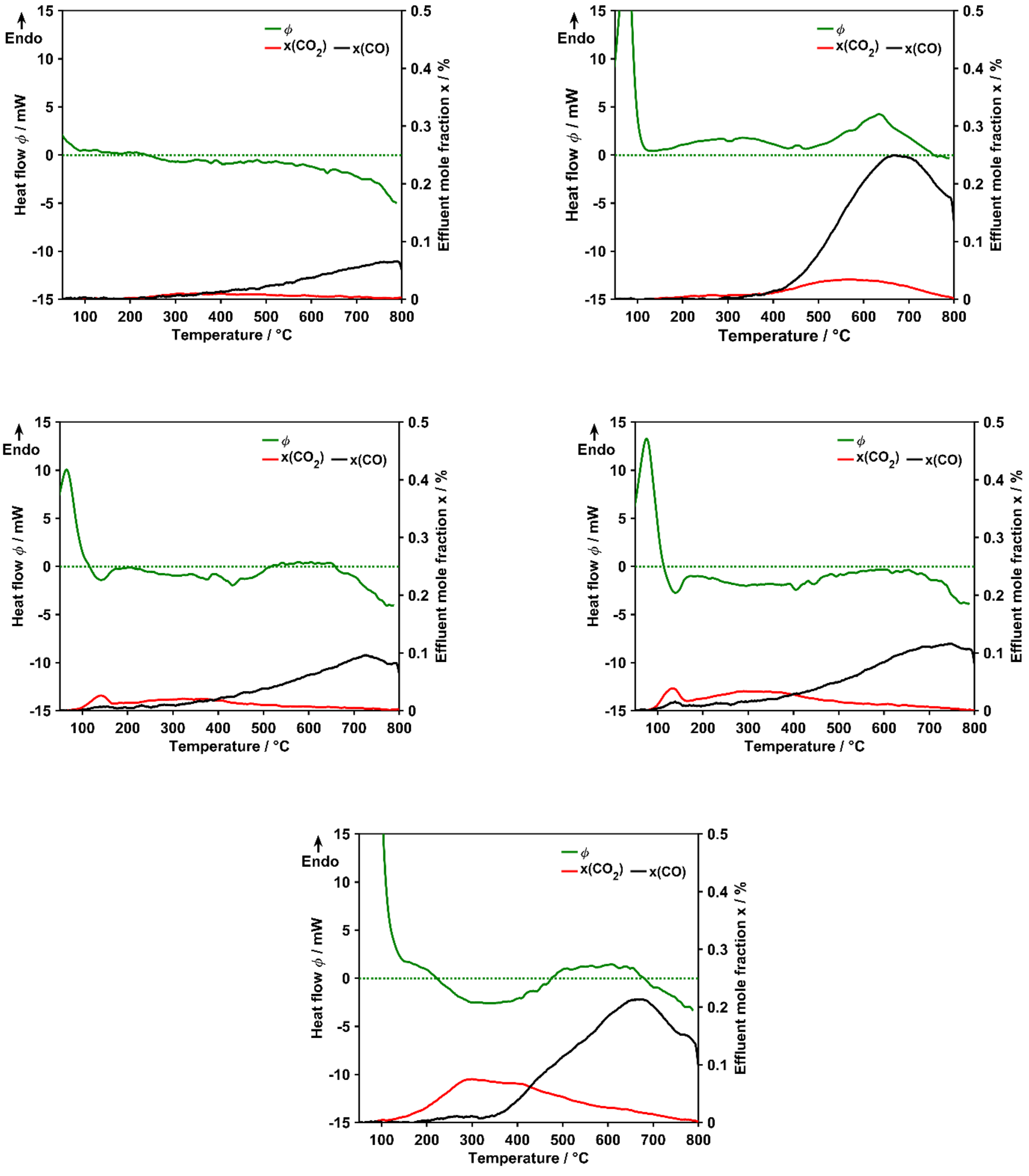

Figure S3. Heat flow and CO_x evolution curves of the less functionalized samples with an O content of up to 25% (top left: O-MH800-O_2-200, top right: O-MH800-O_2-425, center left: O-MH800-0.5-125, center right: O-MH800-1-125, bottom: O-MH800-1-200).

Although the signal intensity of the CO_x TPD-EGA and heat flow curves of the less intensely oxidized chars (Fig. S3) was not sufficient to perform the deconvolution procedure, it is possible to check for consistency based on the OFG assignment (Table 2) and heat flow trends (Table 5) derived for the more extensively functionalized samples:

(a) For O-MH800-O_2-200 there is mainly the high-temperature CO evolution attributed to carbonyls/quinones observed. The heat flow signal which is close to zero at lower temperatures decreases towards the higher temperature end, thus being consistent with a pronounced exothermic effect of carbonyls/quinones.

(b) For O-MH800-O2-425 three different evolving OFGs can be assumed. The CO_x release at lower temperatures indicates the presence of anhydride groups which is in agreement with an overall endothermic effect in this range. At higher temperatures there is a simultaneous release of CO from phenols/ethers and CO_2 from lactones. The larger exothermic heat effect of the latter OFGs is overcompensated by the much larger amount of endothermically decomposing phenols/ethers, resulting in total in a pronounced endothermic effect.

(c) For O-MH800-0.5-125 CO_2 evolution at comparable low temperatures is observed, likely originating from destabilized acids. With increasing temperature, more stable acid groups decompose to additional CO_2, whereas at high temperatures there is significant CO evolution, indicating mainly carbonyls/quinones. The exothermicity is especially pronounced for the destabilized acid groups, but a slight effect is also observed for the more stable ones. Congruent with the high temperature decomposition of carbonyls/quinones, there is again an exothermic effect detected.

(d) For O-MH800-1-125 the qualitative trend is similar to O-MH800-0.5-125. However, the larger concentration of acid groups decomposing to CO_2 leads to an exothermic effect in the same temperature range which is distinctively different from the zero line.

(e) For O-MH800-1-200 there is an increased variety of decomposing OFGs. At lower temperatures, there is the CO_2 evolution typical for acid groups followed by simultaneous CO_2 and CO release indicating the presence of anhydrides. At higher temperatures, there is a slight CO_2 evolution due to lactones as well as a pronounced CO evolution due to phenols/ethers and possibly also carbonyls/quinones. Accordingly, the heat flow is composed of an exothermic effect characteristic for acids, followed by an endothermic effect which results from the dominance of endothermically decomposing anhydrides and phenols/ethers compared to lactones. Finally, there is again a pronounced exothermic effect due to carbonyls/quinones.

Overall, the consistency of the obtained heat effect quantification was evident focusing not only on samples generated by varied temperature and duration of the HNO_3 vapor treatment but also on samples generated by another type of treatment. This is especially important, as the oxidation by molecular oxygen in case of the samples O-MH800-O_2-200 and O-MH800-O_2-425 is more closely related to realistic combustion conditions than the HNO_3 vapor functionalization of the samples used for deconvolution.

S4 Supplementary Deconvolution Data of TPD-EGA and Calorimetric TPD Curves

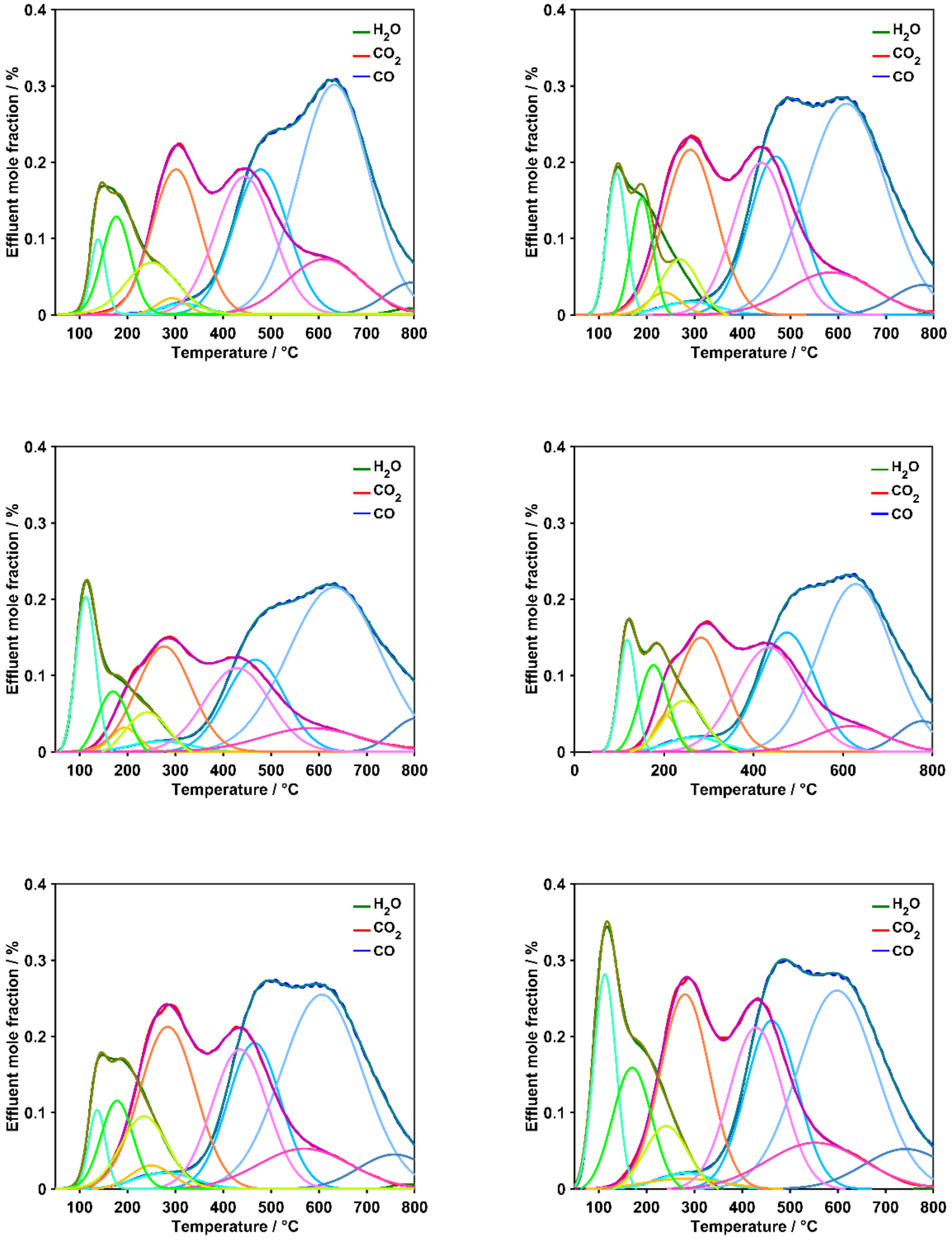

Figure S4. Deconvoluted experimental TPD curves (top left: O-MH800-6-200, top right: O-MH800-6-150, center left: O-MH800-6-125, center right: O-MH800-12-125, bottom left: O-MH800-18-125, O-MH800-24-125) with Gaussian contributions and fitted curves as assigned in Table S2 for the different evolving gases.

Table S2. Assignment of fit contributions in the TPD curves (Fig. S4) for the different evolving gases with increasing thermal stability from top to bottom.

H_2O	CO_2	CO
Phys	CondAc	CondAc
Clus	CarbAc	Anh
Cond	Anh	PheEth
	Lac	CarQui

Table S3. Gaussian fit parameters (area A in % °C, width v and center temperature T_c in °C) obtained by deconvolution of the TPD curves (top: H_2O, center: CO_2, bottom: CO).

Sample	Phys			Clus			Cond		
	A	v	T_c	A	v	T_c	A	v	T_c
O-MH800-6-200	4	31	139	10	60	177	9	107	251
O-MH800-6-150	6	36	133	11	68	176	10	99	241
O-MH800-6-125	11	42	113	7	66	170	7	91	241
O-MH800-12-125	7	38	117	9	69	172	10	107	235
O-MH800-18-125	5	34	137	10	66	178	12	97	235
O-MH800-24-125	17	47	113	17	82	170	10	92	241

Sample	CondAc			CarbAc			Anh			Lac		
	A	v	T_c	A	v	T_c	A	v	T_c	A	v	T_c
O-MH800-6-200	2	62	294	24	101	302	27	21	445	14	157	611
O-MH800-6-150	3	68	237	30	109	291	29	115	439	13	180	585
O-MH800-6-125	2	58	194	20	115	276	19	136	429	8	207	584
O-MH800-12-125	3	51	203	21	110	282	25	147	435	7	159	616
O-MH800-18-125	3	98	254	31	115	282	29	120	434	12	183	580
O-MH800-24-125	3	173	280	33	103	280	30	112	429	16	204	553

Sample	CondAc			CarbAc			Anh			Lac		
	A	v	T_c	A	v	T_c	A	v	T_c	A	v	T_c
O-MH800-6-200	2	91	317	27	114	479	56	149	632	6	118	799
O-MH800-6-150	3	118	291	29	111	469	59	169	617	6	124	777
O-MH800-6-125	2	127	270	19	123	467	52	193	632	6	100	804
O-MH800-12-125	3	121	267	25	128	475	47	169	630	5	100	778
O-MH800-18-125	3	127	278	29	113	467	52	162	609	8	134	755
O-MH800-24-125	3	117	283	30	108	462	53	163	598	11	174	742

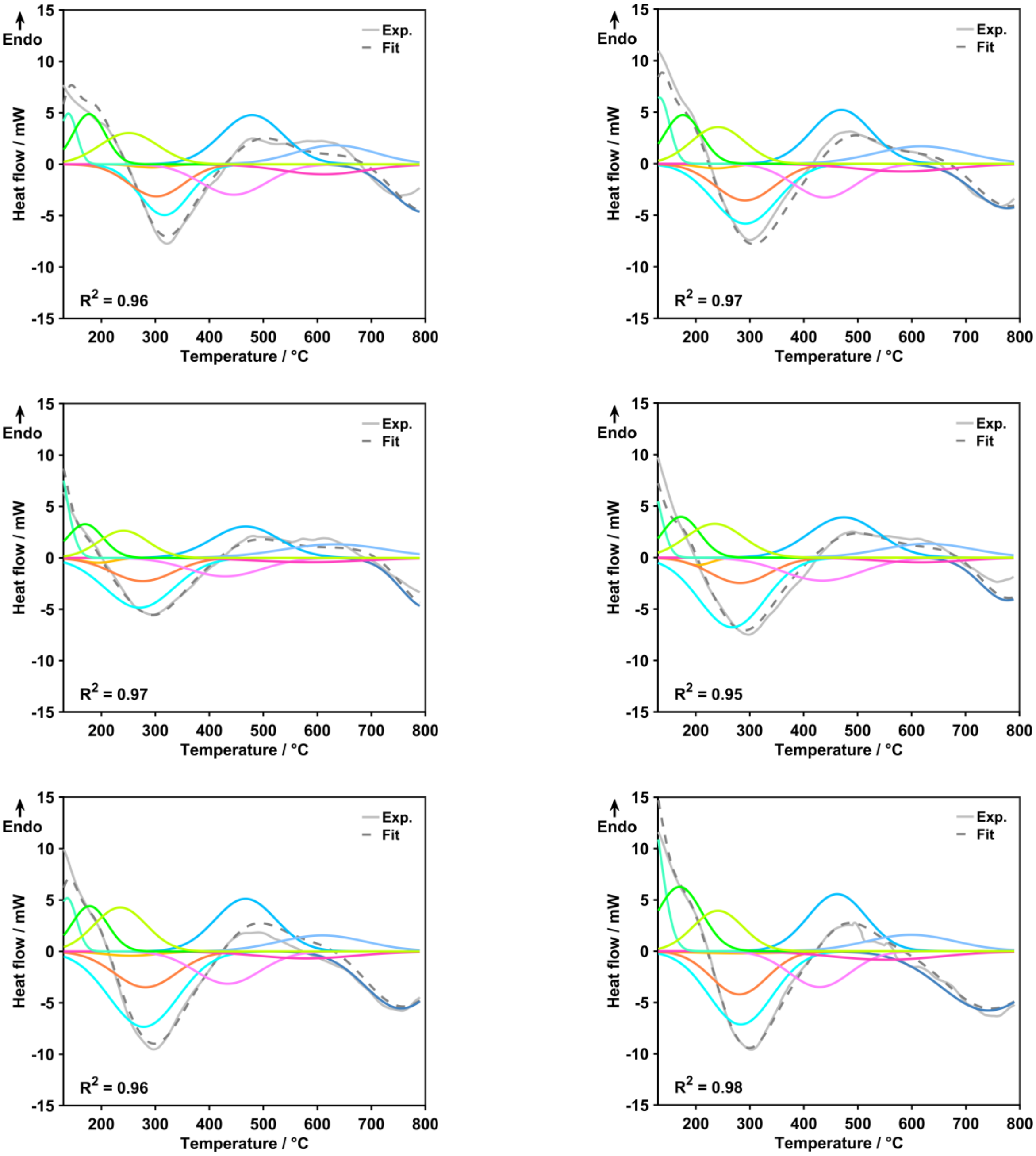

Figure S5. Deconvoluted experimental (Exp.) heat flow curves (top left: O-MH800-6-200, top right: O-MH800-6-150, center left: O-MH800-6-125, center right: O-MH800-12-125, bottom left: O-MH800-18-125, O-MH800-24-125) with Gaussian contributions as assigned in Table S4 for the different OFG decompositions, and fitted curves (Fit) of qualities R^2 determined according to Eq. (4).

Table S4. Assignment of contributions in the heat flow curves (Fig. S5) for the different evolving gases with increasing thermal stability from top to bottom.

H_2O	CO_2	CO
Phys	CondAc	CondAc
Clus	CarbAc	Anh
Cond	Anh	PheEth
	Lac	CarQui

S5 Supplementary Information on the Theoretical Models

S5.1 Computational Details

The DFT calculations were performed using the Turbomole program package[5] version 7.5[6] employing the ridft module. The TPSS functional[7] was chosen in combination with the def2-SVP basis set.[8] The corresponding auxiliary basis set was taken from Eichkorn et al.[9] Furthermore, Grimme's D3 dispersion correction[10] was applied. The construction of the models was performed mostly with Python code using the CGAL (4.2)[11] library for the construction of the hypersurfaces, the pydlpoly code[12] as a molecular mechanics backend for preoptimization after prepositioning of the carbon atoms. In the MD simulations, the char models were equilibrated using ReaxFF as implemented in the ADF software suite (2018.103).[13,14] For the production level calculations, the extended tight binding methods (xTB, GFN2 parameterization)[15] were used, since due to the ReaxFF parameterization the model was even stable at high temperatures and did not decompose. The inertness of the system treated on the ReaxFF level is due to the parameterization and can only be partially overcome by longer simulation times.

S5.2 Figures of the Theoretical Models

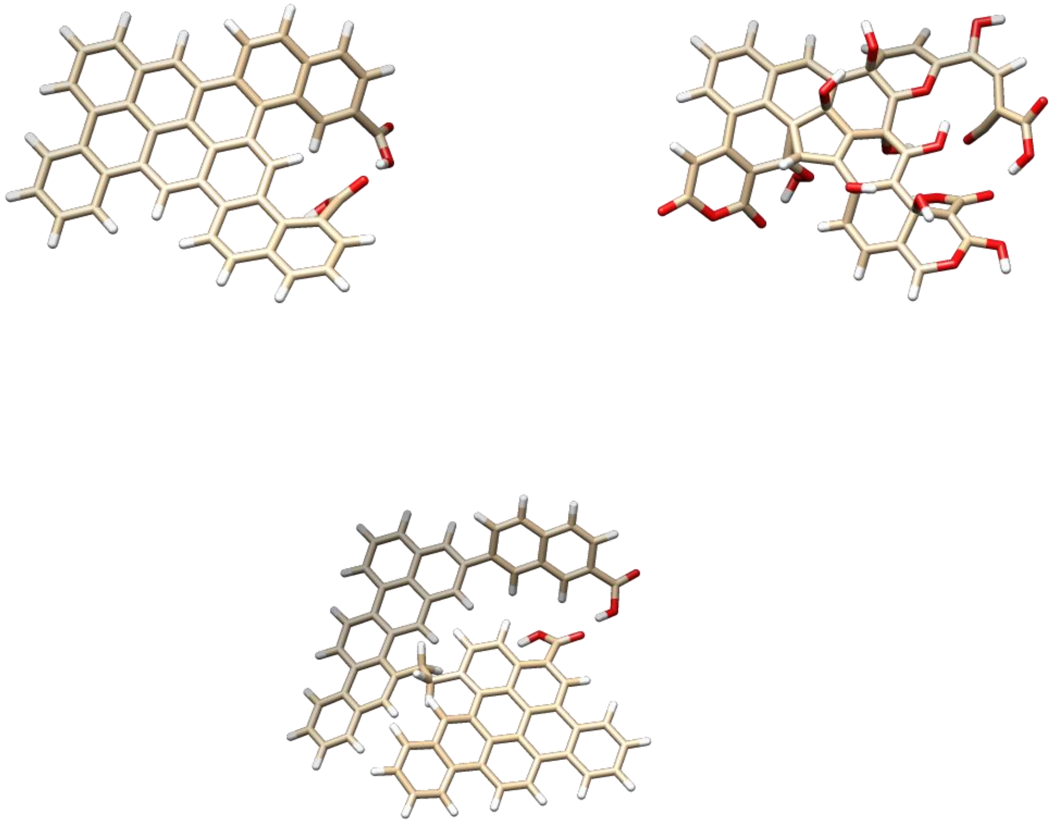

Figure S6. Different models considered in the DFT calculations (top left: minimal OFG incorporation, top right: incorporation of additional OFGs, bottom: two sheets).

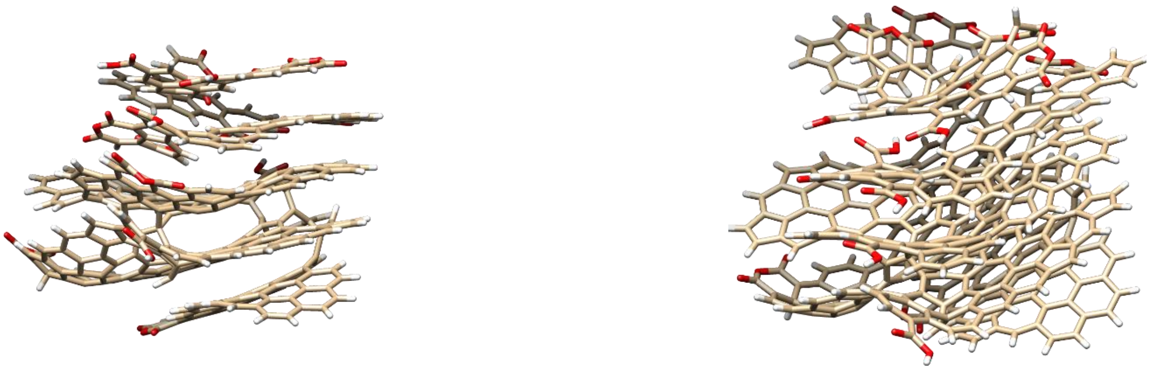

Figure S7. Selected char model used in the MD simulation containing mainly anhydrides as OFGs.

AUTHOR INFORMATION

Corresponding Authors

*E-mail: francesca.cerciello@ruhr-uni-bochum.de; muhler@techem.rub.de

REFERENCES

(1) Gosselink, R. W.; van den Berg, R.; Xia, W.; Muhler, M.; Jong, K. P. de; Bitter, J. H. Gas phase oxidation as a tool to introduce oxygen containing groups on metal-loaded carbon nanofibers. *Carbon* **2012**, *50*, 4424–4431.

(2) Senneca, O.; Salatino, P.; Cortese, L. Assessment of the thermochemistry of oxygen chemisorption and surface oxide desorption during looping combustion of coal char. *Proc. Combust. Inst.* **2013**, *34*, 2787–2793.

(3) Tamargo-Martínez, K.; Villar-Rodil, S.; Martínez-Alonso, A.; Tascón, J. Chemical and structural modifications of carbon nanofibers with different degrees of graphitic order following oxygen plasma treatments. *Mater. Chem. Phys.* **2013**, *138*, 615–622.

(4) Düngen, P.; Schlögl, R.; Heumann, S. Non-linear thermogravimetric mass spectrometry of carbon materials providing direct speciation separation of oxygen functional groups. *Carbon* **2018**, *130*, 614–622.

(5) Balasubramani, S. G.; Chen, G. P.; Coriani, S.; Diedenhofen, M.; Frank, M. S.; Franzke, Y. J.; Furche, F.; Grotjahn, R.; Harding, M. E.; Hättig, C.; Hellweg, A.; Helmich-Paris, B.; Holzer, C.; Huniar, U.; Kaupp, M.; Marefat Khah, A.; Karbalaei Khani, S.; Müller, T.; Mack, F.; Nguyen, B. D.; Parker, S. M.; Perlt, E.; Rappoport, D.; Reiter, K.; Roy, S.; Rückert, M.; Schmitz, G.; Sierka, M.; Tapavicza, E.; Tew, D. P.; van Wüllen, C.; Voora, V. K.; Weigend, F.; Wodyński, A.; Yu, J. M. TURBOMOLE: Modular program suite for ab initio quantum-chemical and condensed-matter simulations. *J. Chem. Phys.* **2020**, *152*, 184107.

(6) University of Karlsruhe and Forschungszentrum Karlsruhe GmbH 1989-2007, TURBOMOLE GmbH since 2007. *TURBOMOLE V7.5*. https://www.turbomole.org/ (accessed 2022-05-22).

(7) Tao, J.; Perdew, J. P.; Staroverov, V. N.; Scuseria, G. E. Climbing the Density Functional Ladder: Nonempirical Meta-Generalized Gradient Approximation Designed for Molecules and Solids. *Phys. Rev. Lett.* **2003**, *91*, 146401.

(8) Schäfer, A.; Horn, H.; Ahlrichs, R. Fully optimized contracted Gaussian basis sets for atoms Li to Kr. *J. Chem. Phys.* **1992**, *97*, 2571–2577.

(9) Eichkorn, K.; Treutler, O.; Öhm, H.; Häser, M.; Ahlrichs, R. Auxiliary basis sets to approximate Coulomb potentials (Chem. Phys. Letters 240 (1995) 283-290). *Chem. Phys. Lett.* **1995**, *242*, 652–660.

(10) Grimme, S.; Antony, J.; Ehrlich, S.; Krieg, H. A consistent and accurate ab initio parametrization of density functional dispersion correction (DFT-D) for the 94 elements H-Pu. J. Chem. Phys. **2010**, *132*, 154104.

(11) The CGAL Project. *CGAL User and Reference Manual*, 4.13rd ed.; CGAL Editorial Board, 2018.

(12) Bureekaew, S.; Amirjalayer, S.; Tafipolsky, M.; Spickermann, C.; Roy, T. K.; Schmid, R. MOF-FF - A flexible first-principles derived force field for metal-organic frameworks. *Phys. Status Solidi B* **2013**, *250*, 1128–1141.

(13) van Duin, A. C. T.; Dasgupta, S.; Lorant, F.; Goddard, W. A. ReaxFF: A Reactive Force Field for Hydrocarbons. *J. Phys. Chem. A* **2001**, *105*, 9396–9409.

(14) Chenoweth, K.; van Duin, A. C. T.; Goddard, W. A. ReaxFF reactive force field for molecular dynamics simulations of hydrocarbon oxidation. *J. Phys. Chem. A* **2008**, *112*, 1040–1053.

(15) Bannwarth, C.; Ehlert, S.; Grimme, S. GFN2-xTB-An Accurate and Broadly Parametrized Self-Consistent Tight-Binding Quantum Chemical Method with Multipole Electrostatics and Density-Dependent Dispersion Contributions. *J. Chem. Theory Comput.* **2019**, *15*, 1652–1671.
